# The effectiveness of regional cooling for paclitaxel-induced peripheral neuropathy

**DOI:** 10.1186/s40780-016-0067-2

**Published:** 2016-11-15

**Authors:** Junya Sato, Megumi Mori, Satoru Nihei, Masumi Kumagai, Satoshi Takeuchi, Masahiro Kashiwaba, Kenzo Kudo

**Affiliations:** 1 Department of Pharmacy, Iwate Medical University Hospital, 19-1 Uchimaru, Morioka, Iwate, 020-8505 Japan; 2 Department of Clinical Pharmaceutics, School of Pharmacy, Iwate Medical University, 2-1-1 Nishitokuta, Yahaba, Iwate, 028-3694 Japan; 3 Department of Nursing, Iwate Medical University Hospital, 19-1 Uchimaru, Morioka, Iwate, 020-8505 Japan; 4 Department of Obstetrics and Gynecology, School of Medicine, Iwate Medical University, 19-1 Uchimaru, Morioka, Iwate, 020-8505 Japan; 5 Department of Breast Surgery, Breastpia Miyazaki Hospital, 2-112-1 Maruyama, Miyazaki, Miyazaki, 880-0052 Japan

**Keywords:** Regional cooling, Peripheral neuropathy, Paclitaxel, Gynecologic cancer, Chemotherapy

## Abstract

**Background:**

There are currently no promising therapies available to treat or prevent peripheral neuropathy (PN) induced by anticancer drugs in a cumulative dose-dependent manner. In this study, we investigated the efficacy of regional cooling of hands and feet in preventing paclitaxel (PTX)-induced PN.

**Methods:**

Patients with gynecologic cancer who received a tri-weekly cycle of chemotherapy including PTX at doses of 150–175 mg/m^2^ were included in this study. Regional cooling was performed by covering patient hands and feet with cold insulators during PTX administration (regional cooling group). The primary end-point was ≥grade 2 PN evaluated by the Common Terminology Criteria for Adverse Events (CTCAE) v4.0. The secondary end-points were the frequency of PN therapeutic drug use, PTX dose reduction due to PN, and adverse events due to regional cooling. The efficacy of regional cooling was compared with data retrospectively extracted from the medical records of patients who did not receive regional cooling (control group). All end-points were evaluated for up to six cycles.

**Results:**

There were 40 and 142 patients in the regional cooling and control groups, respectively. As a primary end-point, incidences of ≥grade 2 PN in the fourth to sixth cycles were significantly lower than that in the cooling group (5.0–9.1 % vs. 19.8–31.6 %, *p* < 0.05 after the fourth cycle and *p* < 0.01 after the fifth cycle). Among secondary end-points, neither the use of PN therapeutic drugs nor the PTX dose reduction due to PN were significantly lower in the cooling group than in the control group (27.5 vs. 36.6 %, *p* = 0.378 and 5.0 vs. 3.5 %, *p* = 0.645, respectively). There were no serious regional cooling-associated adverse events such as frostbite.

**Conclusions:**

Regional cooling of hands and feet during PTX administration might have good effectiveness and tolerability, suggesting this approach as a potentially effective supportive care to prevent PTX-induced PN.

**Trial registration:**

The trial approval number in the institution; H25-26. Registered 5 June 2014.

## Background

Peripheral neuropathy (PN) is caused by the administration of anticancer drugs such as taxane, platinum, and vinca alkaloid. The symptoms of PN begin as glove/sock-type hypesthesia, hyperesthesia, or dysesthesia of peripheral areas, such as the hands and feet [1]. These symptoms spread and increase in severity in a cumulative dose-dependent manner. Activities of daily living (ADLs), such as writing, getting dressed, and eating, are gradually affected. When PN reaches a severe stage, motor nerve disorders of the hands and feet inhibit some ADLs, such as walking; these can persist for a long time, greatly impairing the quality of life [2, 3]. Reduction or discontinuation of PN-inducing anticancer drugs based on appropriate evaluation and early detection is an important component of the management of chemotherapy.

One of anticancer drug that induces PN is paclitaxel (PTX). In several phase III studies using tri-weekly TC therapy [PTX: 175–180 mg/m^2^ and carboplatin (CBDCA): AUC = 5–6] and tri-weekly TP therapy [PTX: 175 mg/m^2^ and cisplatin (CDDP): 75 mg/m^2^] as standard chemotherapy regimens for gynecologic cancer, the frequencies of grade ≥3 PN neurosensory and neuromotor symptoms were in the range of 6–14 % and 2–7 %, respectively [4–7].

High plasma PTX concentrations of 0.05 μmol/L or more have been reported to be associated with the development of PN [8]. This previously reported high PTX blood concentration was observed for 24 h in the tri-weekly PTX regimen (including more than 175 mg/m^2^ PTX) [9]. Therefore, a decrease in the plasma PTX concentration in peripheral regions might prevent to develop PN.

Regional cooling is one of the methods used to reduce peripheral exposure to anticancer drugs in clinical practice. However, the effect of regional cooling in preventing chemotherapy-induced PN has not been investigated. In this study, we investigated the effect of hand and foot cooling on the development of PN in patients who received PTX including chemotherapy for gynecologic cancer.

## Methods

### Patients

Patients with gynecologic cancer (ovarian, cervical, or endometrial cancer) who received chemotherapy including PTX at doses of 150–175 mg/m^2^ for 3 h every 3 weeks, concomitant with CBDCA or CDDP, at Iwate Medical University Hospital between July 2014 and November 2015 were eligible for intervention in this study. Patients with preexisting subjective PN symptoms prior to chemotherapy initiation and those with potential PN due to prior chemotherapy regimens including taxane or platinum by a recurrence of disease within 1 year were excluded. Patients with new diagnosis and those with cancer recurrence more than 1 year after last chemotherapy received regional cooling (regional cooling group) after consent for participation in this study was obtained. Patients were not randomized during the intervention period. The effect of regional cooling was compared with historical control data which was retrospectively extracted from the medical records of patients who received similar chemotherapy regimens during the period before regional cooling intervention (April 2011–June 2014, control group). In this historical control cohort, patients with recurrent disease within 1 year prior to enrollment in this study and those with subjective PN symptoms before chemotherapy initiation were excluded. As an exclusion criteria for the analysis, patients who discontinued chemotherapy early during treatment (<4 cycles) in both the regional cooling and control groups were excluded from analysis. Furthermore, given that diabetes, current neuropathy, and past platinum use were reported as risk factors for PN [10], patients with a medical history of diabetes, rheumatoid arthritis, lymphedema, and/or treatment for PN before chemotherapy initiation were also excluded from the analysis.

### Chemotherapy regimen

The details of each chemotherapy regimen were as follows: TC ± bevacizumab (BV); PTX 175 mg/m^2^ + CBDCA AUC = 6 ± BV 15 mg/kg, TP; PTX 175 mg/m^2^ + CDDP 75 mg/m^2^, TEC; PTX 150 mg/m^2^ + Epirubicin 50 mg/m^2^ + CBDCA AUC = 5; and TAC; PTX 150 mg/m^2^ + Adriamycin 45 mg/m^2^ + CBDCA, AUC = 5.

### Method of regional cooling

**Fig. 1:**
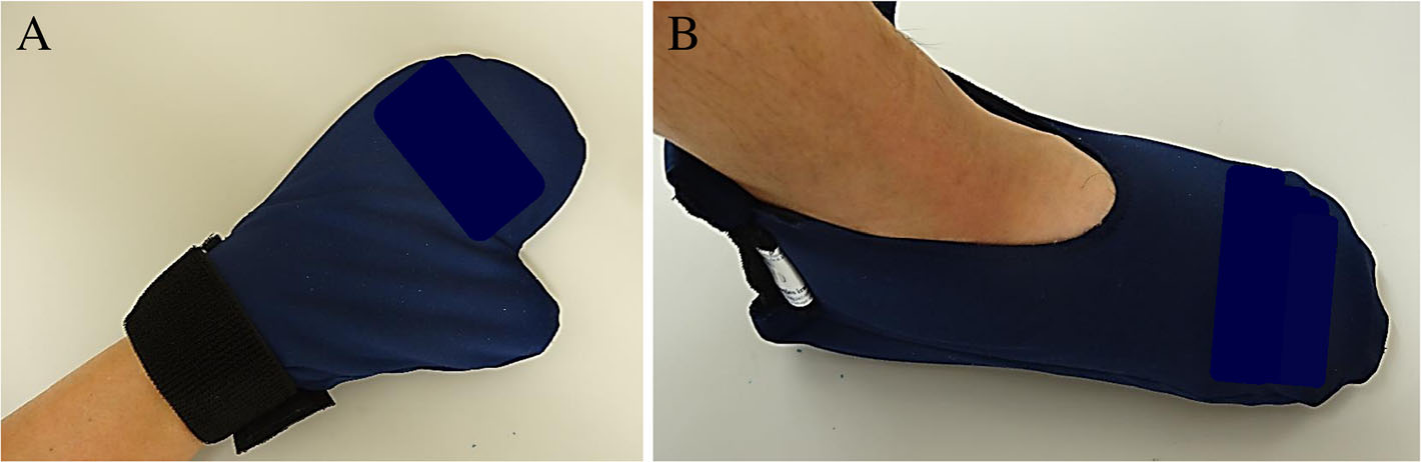
Cold insulators for hands (**a**) and feet (**b**). Figure 1 indicated cold insulators used in this study. The pre-cooled insulators were fitted on both hands and feet. The insulators were changed hourly

Regional cooling was performed using cold insulators as shown in Fig. 1 (Elasto-Gel™ mitts for hands: TM7008, and slippers for feet: SL3000; Southwest Technologies, Inc., North Kansas City, MO, USA), which were pre-cooled to −30 °C before use. The insulators were fitted on both hands and feet for 3 h under PTX administration. The cold insulators were changed hourly.

### Evaluation criteria and timing

The severity of PN was evaluated by a physician or a pharmacist using the Common Terminology Criteria for Adverse Events (CTCAE) v4.0 before the start of each chemotherapy cycle as well as 3 weeks after the last chemotherapy in the regional cooling group. Retrospective data collected from the medical records of the control group also contained information on PN symptoms from time points comparable to those of the cooling group. Evaluation for PN symptoms was performed for up to six cycles.

### Primary and secondary end-point

The primary end-point was ≥grade 2 PN. The secondary end-points were the frequency of PN therapeutic drugs use (with therapeutics including pregabalin, gabapentin, duloxetine, Gosha-jinki-gan, mecobalamin, tramadol hydrochloride, and opioids), PTX dose reduction due to PN, and adverse events of regional cooling.

### Statistical analysis

Background information of patients such as age, number of chemotherapy cycles, body surface area, initial PTX dose, cumulative PTX dose, and cumulative platinum dose were presented as means ± standard deviation, and differences were analyzed by unpaired *t* test. Differences in type of cancer, regimen, and the presence of concomitant platinum use were analyzed by the chi-square test. The incidence of ≥grade 2 PN, PN therapeutic drug use, and PTX reduction due to PN were analyzed by the chi-square test. Hazard ratios of less than 5 % were considered statistically significant. Statistical analyses were performed using Excel® Statistics 2012 software (Social Information Service, Tokyo, Japan).

## Results

### Patient background

**Fig. 2:**
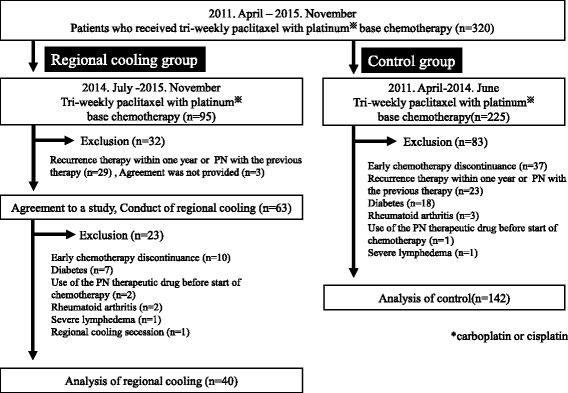
Consort diagram in this study. Figure 2 indicated the consort diagram of this study. Sixty three patients agreed to the participation in the study and received regional cooling. Forty patients who were able to continue chemotherapy were analyzed. As the control group, 225 patients who received similar chemotherapy in the period before intervention of the regional cooling were retrospectively extracted from medical chart. One hundred-forty two patients were analyzed

A consort diagram for this study is shown in Fig. 2. There were 95 patients who received chemotherapy including tri-weekly PTX concomitant with CBDCA or CDDP from July 2014 to November 2015. Among these, 32 patients were excluded due to treatment for recurrence within one year or PN resulting from previous chemotherapy (*n* = 29) or due to lack of sufficient information (*n* = 3). Therefore, 63 patients were started on regional cooling with chemotherapy initiation. Of these, 23 patients were excluded from the final analysis due to early chemotherapy discontinuance (<4 cycles, *n* = 10), history of diabetes (*n* = 7), use of the PN therapeutic drug before chemotherapy initiation (*n* = 2), history of rheumatoid arthritis (*n* = 2), severe lymphedema (*n* = 1), and regional cooling secession (*n* = 1); therefore, a total of 40 patients in the regional cooling group were included in the final analysis.

In the control group, there were 225 patients who received chemotherapy including tri-weekly PTX concomitant with CBDCA or CDDP from April 2011 to June 2014. Among these, 83 patients were excluded due to early chemotherapy discontinuance (<4 cycles, *n* = 37), treatment for recurrence within one year or PN due to previous chemotherapy (*n* = 23), history of diabetes (*n* = 18), rheumatoid arthritis (*n* = 3), use of the PN therapeutic drug before chemotherapy initiation (*n* =1), and severe lymphedema (*n* = 1); therefore, a total of 142 patients in the control group were included in the final analysis.

Background characteristics of patients are represented in Table 1. There were significant differences in the distribution of regimens and use of concomitant platinum between the two groups (*p* = 0.004 and *p* = 0.006, respectively). However, there were no significant differences in age, cancer type, number of chemotherapy cycles, body surface area, PTX, and platinum dose between the two groups.

**Table 1:** Patients background

	Control group (*n* = 142)	Cooling group (*n* = 40)	*P*-value
Age (min-max)	59.7 ± 11.5 (35–84)	56.5 ± 10.4 (32–77)	0.144
Cancer type	Ovarian (*n* = 80) Cervical (*n* = 14) Endometrial (*n* = 48)	Ovarian (*n* = 23) Cervical (*n* = 9) Endometrial (*n* = 8)	0.053
Regimen	TC ± BV (*n* = 137) TP (*n* = 2) TAC or TEC (*n* = 3)	TC ± BV (*n* = 35) TP (*n* = 5)	0.004
Concomitant platinum	CBDCA (*n* = 140) CDDP (*n* = 2)	CBDCA (*n* = 35) CDDP (*n* = 5)	0.006
Cycle	5.9 ± 0.4	5.9 ± 0.4	0.624
Body-surface area (m2)	1.526 ± 0.151	1.483 ± 0.127	0.233
Initial PTX dose (mg/m2)	170.2 ± 6.9	171.2 ± 8.4	0.944
Cumulative PTX dose (mg/m2)	997.4 ± 78.9	1010.1 ± 95.1	0.483
Cumulative Platinum dose (mg/m2)	CBDCA; 2291.3 ± 391.7 CDDP; 324.8 ± 34.3	CBDCA; 2332.2 ± 301.6 CDDP; 295.7 ± 5.5	0.565 0.311

The value of age, cycle, body-surface area, initial PTX dose, cumulative PTX dose, and cumulative platinum dose were indicated as means ± standard deviation and its *p* values were calculated by unpaired *t*-test. The *p* value of cancer type, regimen, concomitant platinum were calculated by chi-square test. *PTX* paclitaxel, *CBDCA* carboplatin, *CDDP* cisplatin, *TC* ± *BV* bevacizumab ± paclitaxel + carboplatin, *TP* paclitaxel + cisplatin, *TAC* paclitaxel + doxorubicin + carboplatin, *TEC* paclitaxel + epirubicin + carboplatin

### Primary end-point

**Fig. 3:**
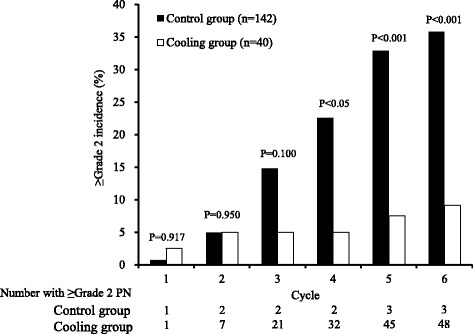
Effect of the regional cooling on the incidence of Grade ≥2 PN. Figure 3 indicated the incidence of Grade ≥2 PN. The *black bar* indicated the PN incidence of control group (*n* = 142) in each cycle. The *white bar* indicated the PN incidence of regional cooling group (*n* = 40) in each cycle. The comparison of the both groups in each cycle performed by a chi-square test

The incidence rate of ≥grade 2 PN in the regional cooling group (5.0–9.1 %) was significantly lower than that observed in the control group (22.5–35.8 %, *p* < 0.05 in the fourth cycle and *p* < 0.01 after the fifth cycle, Fig. 3).

### Secondary end-points

There was no significant difference in the frequency of PN therapeutic drug use in the regional cooling group than in the control group (27.5 vs. 36.6 %, *P* = 0.378, Table 2). Specifically, the following medications were prescribed for PN therapeutic drugs (There is, with some patients receiving combination therapy: Gosha-jinki-gan (*n* = 7), mecobalamin (*n* = 3), pregabalin (*n* = 1), oxycodone hydrochloride (*n* = 1), and duloxetine hydrochloride (*n* = 1) in the regional cooling group; pregabalin (*n* = 22), Gosha-jinki-gan (*n* = 22), mecobalamin (*n* = 10), and tramadol hydrochloride (*n* = 1) in the control group. There was also no significant difference in the frequency of PTX dose reduction due to PN between the two groups (5.0 vs. 3.5 %, *p* = 0.645, Table 2). Severe adverse events related to regional cooling such as frostbite were not observed. However, one patient discontinued regional cooling halfway through a cycle (at three cycle) because of cold-induced pain.

**Table 2:** Effect of the regional cooling on the prescription of the PN therapeutic drug and PTX doses reduction due to PN

Cycle	Control group (*n* = 142)	Regional cooling group (*n* = 40)	*p*-value
Prescription of the PN therapeutic drug※	52 (36.6 %)	11 (27.5 %)	*P* = 0.378
PTX doses reduction due to PN	5 (3.5 %)	2 (5.0 %)	*P* = 0.645

*PN* Peripheral neuropathy. The *p* value indicated hazard ratio of the chi-square test

※pregabalin, duloxetine hydrochloride, mecobalamin, gosha-jinki-gan, tramadol, oxycodone

## Discussion

PN induced by PTX is thought to be reversible because axons are reproduced upon discontinuation of the drug. This is different for CDDP-induced irreversible PN because of direct damage of the perikaryon [11, 12]. Most patients that were administered PTX suffer PN for a long time. The PTX-induced PN develops depending on increasing once dose and cumulative dose of PTX, shortening of PTX infusion time. For example, in a controlled trial using a fixed dose intensity of PTX in ovarian cancer, there was a significantly higher incidence of grade 3 PN in patients administered 200 mg/m^2^/3 weeks than in those administered 67 mg/m^2^/week (29 vs. 11 %, *P* < 0.001) [13]. In addition, in a systematic review, PN was reported to be more common upon the 3 h administration of PTX than upon 24 h administration [14]. The average cumulative dose at which grade ≥2 PN developed was reported to be 715 mg/m^2^ when 175 mg/m^2^ PTX was given every 3 weeks in patients with metastatic breast cancer [15]. For gynecologic cancer, TC regimen including high-dose PTX administration was performed every 3 weeks as standard chemotherapy. It is important that these TC regimens generally be completed within four to six cycles [16]. As the cumulative PTX dose in this clinical practice is 700 mg/m^2^ or more, PN is an unavoidable adverse event.

Few drugs have been used to prevent or relieve chemotherapy-induced PN. Classically, antiepileptics or antidepressants such as carbamazepine and amitriptyline have been administered, but recently gabapentin and duloxetine have been widely used [17–19]. However, these drugs do not seem to provide much benefit because of their low efficacy and serious adverse effects such as somnolence. Only duloxetine was moderately recommended for therapeutic use for chemotherapy-induced PN in the guidelines of the American Society of Clinical Oncology [20]. However, taxane-induced PN might be more difficult to than oxaliplatin-induced PN, even if duloxetine is used [21]. Therefore, non-pharmacological therapy with therapeutic efficacy and few adverse effects was expected for chemotherapy-induced PN.

In this study, we investigated the efficacy of regional cooling as a potential non-pharmacological prophylactic therapy for PTX-induced PN. Our results showed that the incidence rates of ≥grade 2 PN were decreased with regional cooling. In particular, there was significantly better cooling efficiency during and after the fourth cycle, at which point PN became severe. Unfortunately, the prescription rates of therapeutic drugs for PN or the frequency of PTX dose reduction due to PN were not significantly lower in the regional cooling group. However, it was thought that inhibition of ≥grade 2 PN by regional cooling was not likely due to the use of these relief drugs and dose reduction of PTX.

The prevention of PN by regional cooling might result from a decrease in regional PTX exposure. In a previous study, experimental cooling of the palms of healthy subjects to 19 °C led to a 53–60 % decrease in blood flow to the palms [22]. Cooling mitts and slippers used in this study decreased the surface temperature of hands and feet by 10–20 °C, which was maintained for up to 1 h in a small number of healthy subjects as determined by thermography. Subsequent studies have successfully reported the use of regional cooling as a supportive modality to relieve adverse effects associated with anti-cancer drugs such as hand and foot cooling for docetaxel-induced nail toxicity [23] and hand-foot syndrome due to pegylated liposomal doxorubicin [24], cool cap for alopecia [25], and cryotherapy with intraoral ice chips for 5-fluorouracil- or melphalan-induced oral mucositis [26, 27]. A similar mechanism might be at play in the protective effect of regional cooling against PN.

The following limitations should be considered during the interpretation of the present study. First, there were differences in regimens and the use of concomitant platinum between the control and regional cooling groups. In majority of the cases, the etiology of PN was considered to be due to PTX; however, platinum might also have contributed to the development of PN in some patients. Specifically, PN develops at cumulative CDDP doses of 250–500 mg/m^2^ [28]. Seven patients in this study were administered CDDP, with a mean cumulative dose of 435 mg/m^2^, and two of these patients developed ≥grade 2 PN. Conversely, CBDCA was reported to have 4–6 % lower incidence of PN than CDDP [29], and the difference in the frequency of PN induction by different platinum drugs might have influenced the results. Another limitation of the present study was the evaluation method, which was conducted by a medical professional, which might have led to a lack of objectivity in evaluation of patients exhibiting low-level PN symptoms [30]. It was possible that the focus of the medical professional was on the recognition of potential PN symptoms during evaluation of patients in the regional cooling group who were enrolled in the intervention, leading to more frequent identification of PN symptoms in this group than in the control group. The regional cooling intervention group was not masked. Given that regional cooling was predicted to be effective by the evaluator, PN might be underestimated, leading to a bias during evaluation. Future investigation with modified designs such as cooling only specific hand and foot in the same patient should resolve this concern. One of the two previous studies investigating docetaxel-associated nail toxicity using a similar design found that the incidence of ≥grade 1 fingernail toxicity was significantly lower in the cooled hand than in the non-cooled hand (11 vs. 51 %, *p* < 0.001) [31]. The outcome of the other study examining toenail toxicity was similar (0 vs. 21 %, *p* = 0.002) [23]. While a similar study design would resolve potential bias, such an approach inadvertently would lead to ethical concerns by allowing for the development of PN on one hand and foot to examine the efficacy of the intervention in the remaining hand and foot.

The clinical evidence of this study was weak as it was not a prospective randomize-controlled study. Therefore, the efficacy of regional cooling for PN should be investigated by future prospective randomized comparison with a non-cooling group in future studies.

## Conclusions

This study was the first to suggest that regional cooling of the hands and feet are effective for the prophylaxis of PTX-induced PN. Regional cooling might be well-tolerated supportive care with few serious adverse effects because it is a non-pharmacological therapy. The efficacy of regional cooling should be further investigated by a randomized comparison.

## Abbreviations

ADLs: Activities of daily living
AUC: Area under the blood concentration-time urve
BV: Bevacizumab
CBDCA: Carboplatin
CDDP: Cisplatin
CTCAE: Common terminology criteria for adverse events
PN: Peripheral neuropathy
PTX: Paclitaxel
